# Rapid Nanoparticle-Mediated Monitoring of Bacterial Metabolic Activity and Assessment of Antimicrobial Susceptibility in Blood with Magnetic Relaxation

**DOI:** 10.1371/journal.pone.0003253

**Published:** 2008-09-23

**Authors:** Charalambos Kaittanis, Sudip Nath, J. Manuel Perez

**Affiliations:** 1 Nanoscience Technology Center, University of Central Florida, Orlando, Florida, United States of America; 2 Burnett School of Biomedical Sciences, University of Central Florida, Orlando, Florida, United States of America; 3 Department of Chemistry, University of Central Florida, Orlando, Florida, United States of America; Instituto de Tecnologia Química e Biológica, Portugal

## Abstract

Considering the increased incidence of bacterial infections and the emergence of multidrug resistant bacteria at the global level, we designed superparamagnetic iron oxide nanoparticles as nanosensors for the assessment of antimicrobial susceptibility through magnetic relaxation. In this report, we demonstrate that iron oxide nanosensors, either dextran-coated supplemented with Con A or silica-coated conjugated directly to Con A, can be used for the fast (1) quantification of polysaccharides, (2) assessment of metabolic activity and (3) determination of antimicrobial susceptibility in blood. The use of these polysaccharide nanosensors in the determination of antimicrobial susceptibility in the clinic or the field, and the utilization of these nanoprobes in pharmaceutical R&D are anticipated.

## Introduction

In the last 20 years, there has been a dramatic increase in the emergence of antibiotic-resistant bacteria, leading to elevated bacterial pathogenesis at the global level [1], [2]. In particular, the emergence of drug resistant strains of *Mycobacterium tuberculosis* (MDR- and XDR-TB) and the increased occurrence of methicillin-resistant *Staphylococcus aureus* (MRSA) infections, indicate that drug resistance is a major public health problem [1], [2]. Recent reports indicate that in the United States MRSA infections cause more deaths than HIV/AIDS, whereas the development of new antibiotics has significantly slowed down since the early 1990s [1], 2, 3. Therefore, developing sensitive and cost-efficient detection systems that can quickly identify if (1) a particular bacterium is resistant to antibiotics, (2) the pharmacological agents which the microorganism is susceptible to and (3) the agents' appropriate effective dosage is vital for successful treatment and prevention of epidemics. Traditionally, the determination of antimicrobial susceptibility to antibiotics is facilitated after isolation of the microorganism and examination of its growth in media containing various antimicrobial agents, in a process that can take up to 48 hours [4], [5]. Hence, developing fast and accurate antimicrobial susceptibility assays is important for the clinic and the pharmaceutical industry.

Nanotechnology offers a unique alternative for the development of detection methods for bacterial targets that require less preparation time and smaller sample volumes, while offering enhanced sensitivity and faster detection kinetics [6], [7], [8], [9], [10]. In particular, nanosensors that not only detect the presence of a particular pathogenic agent directly, but can also make an indirect detection through the assessment of the pathogen's metabolic activity, e.g., via the monitoring of the rate of consumption of nutrients in solution, would be of great utility. The development of such nanosensors will be of significant importance, because they will be able to interrogate whether the pathogen is still metabolically active and reproducing in the presence of a particular antibiotic. Currently, most contemporary nanoparticle-based immunoassays cannot distinguish between metabolically active and dead pathogens [10], [11]. Recently, a novel gold nanoparticle-based method for the assessment of bacterial susceptibility via surface plasmon resonance shifts was reported [12]. The main drawbacks of this technique are the small, although significant, changes in the surface plasmon band and the assay's inability to work in turbid or opaque media, due to the media's strong scattering and absorbance that mask the nanoparticles' plasmonic band. Hence, as some microorganisms, like *Staphylococcus epidermidis* and *Neisseria* spp. among many others, can be present in the blood of infected patients (septicemia), requiring isolation and growth in blood- containing media, it is vital to have clinical diagnostic assays for the assessment of antimicrobial susceptibility in blood [13], [14].

**Figure 1:**
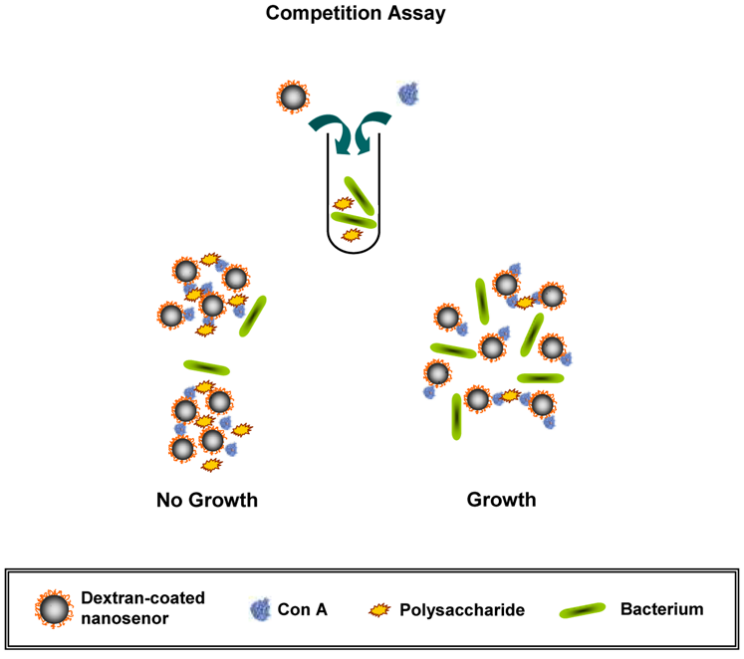
Proposed model for the assessment of antimicrobial susceptibility using dextran-coated polysaccharide nanosensors and Concanavalin A (ConA). In this competition assay, the dextran on the surface of the iron oxide nanoparticles and the starch in solution compete for binding to Con A. This results in changes in the degree of Con-A induced magnetic nanoparticle clustering upon bacterial metabolic uptake of starch.

Consequently, we reasoned that a more robust system, which quickly determines bacterial susceptibility independent of the solution's optical properties, could be developed using magnetic nanosensors and detection via water relaxation [15]. According to the literature, it is widely acknowledged that a major benefit of using magnetic relaxation methods is that molecular detection can be achieved in opaque media, such as cell lysates, tissue extracts and complex biological fluids, notably blood, with high specificity and sensitivity [10], [15], [16]. Therefore, we hypothesized that bacterial-susceptibility-monitoring nanosensors could be designed to differentially respond to the presence of various concentrations of nutrients, such as complex carbohydrates (e.g. starch). Although superparamagnetic nanoparticles have been used as magnetic relaxation sensors for the detection of various targets [10], [15], [16], [17], [18], [19], [20], [21], [22], these nanoprobes have not been previously utilized for the detection of metabolic activity, which might lead to the potential development of nanosensors capable of determining antimicrobial susceptibility in complex media. The polysaccharide nanosensors' clustering should result in a significant change in the spin-spin relaxation time (T2) of the solution's water protons, facilitating the reliable identification of effective antimicrobial agents. This can be achieved using dextran-coated iron oxide nanoparticles along with a protein with high affinity to carbohydrates, such as Concanavalin A (ConA) [23], [24], in a competition assay. Specifically, we hypothesized that upon Con A-induced clustering, the dextran-coated iron oxide nanoparticles can differentially respond to the polysaccharide levels associated with bacterial metabolism and growth. Hence, the higher the rate of bacterial metabolic activity, the fewer amount of the available polysaccharides (such as starch) would be, resulting in prominent changes in the sample's ΔΤ2 when compared to those of the sterile medium (Figure 1).

## Results

### Polysaccharide quantification and bacterial metabolic activity monitoring

**Figure 2:**
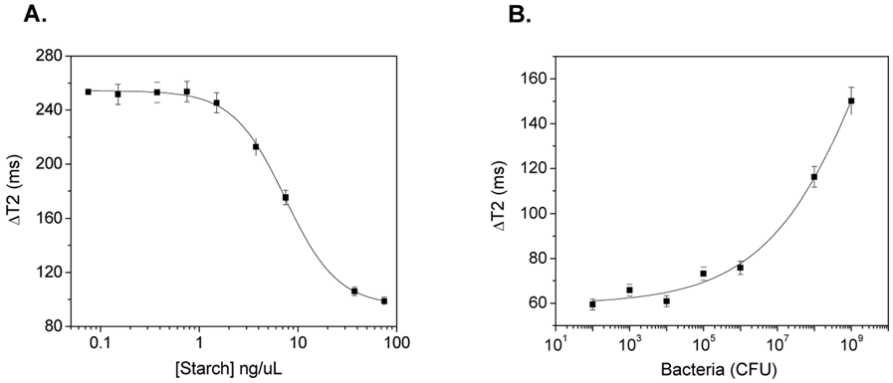
Nanoparticle-mediated sensing of polysaccharide levels and monitoring of bacterial metabolic activity. (A) Quantification of starch in sterile MH broth, and (B) determination of starch consumption due to bacterial metabolism in MH broth, using the dextran-coated polysaccharide nanosensors.

In our first set of experiment, we investigated if our dextran-coated iron oxide nanoparticles (d = ∼150 nm, R2 = 300 mM^−1^s^−1^) were responsive to changes in starch concentration upon bacterial growth. Initial studies in phosphate buffered saline showed that the nanosensors quickly clustered after addition of Con A, (Figure S1) forming stable nanoassemblies that induced prominent changes in the T2 within an hour (Figure S2). During initial optimization studies, we found that a Con A concentration of 1 µg/µL and a nanoparticle solution with a concentration of 0.02 µg Fe/µL provided optimum results (Figure S3). Thus, we used this iron concentration in all of our experiments. Lower iron concentrations increased the error in the measurement, whereas higher concentrations sacrificed sensitivity and detection kinetics, requiring higher concentrations of Con A. Next, we assessed the capability of our nanosensors to sense variations in MH broth's starch concentration. In these experiments we added our nanosensors (0.02 µg Fe/µL) to solutions containing increasing amounts of starch in MH broth and Con-A (1 µg/µL). We anticipated that in this competition assay and at low concentrations of starch, the Con A would be able to cluster the dextran-coated magnetic nanosensors resulting in prominent changes in T2. However, as the amount of starch increases, smaller changes in T2 are expected, as Con A would primarily bind to starch. Results showed that after a 30-minute-long incubation at room temperature, a dose-dependent response curve was obtained (*R* ^2^ = 0.99), demonstrating that the changes in T2 were inversely proportional to the starch concentration, in accordance with our hypothesis (Figure 2A). Notably, the dextran-coated polysaccharide nanosensors were able to quantify starch from concentrations of 77.5 ng/µl, which is the initial starch concentration of a typical MH broth, to 1.5 ng/µL.

We then examined if these polysaccharide nanoprobes can respond to changes in the starch concentration of MH broth (initial starch concentration = 77.5 ng/µl) due to bacterial metabolic activity. In these experiments, magnetic nanoprobe solutions (0.02 µg Fe/µL) containing aliquots of increasing amounts of *E. coli* were incubated with Con A. Results showed that upon addition of Con A, a dose-dependent response was observed (*R* ^2^ = 0.99), after a 30-minute incubation at room temperature (Figure 2B). The higher the bacterial population (expressed in Colony Forming Units, CFU) the larger the changes in T2 (ΔΤ2). This is attributed to the fact that higher bacterial populations utilize more amounts of starch via their metabolic activities, resulting in a concomitant reduction of the media's carbohydrates. As a result, addition of Con A promotes the formation of extensive nanoassemblies, as expected. Notably, even at low bacterial populations (10^2^–10^4^ CFUs) significant changes in T2 were detected. Hence, this demonstrates the high sensitivity of the nanosensors in detecting bacterial metabolic activity, even in the presence of as few as 10^2^ CFUs. This is of grave importance in order to prevent septicemia, as very few bacteria in circulation may cause systemic infection leading to death. In control experiments we found that in the absence of Con A, the presence of increasing amount of bacteria does not affect the T2 of the nanoparticle solution. Specifically, all samples containing either sterile media or different bacterial loads exhibited similar T2 values, suggesting that the presence of bacteria does not alter the spin-spin relaxation times of the non-assembled state (Figure S4). To further corroborate our results, we used heat-inactivated bacteria to inhibit their metabolic activity. Consequently, nominal differences were observed between the samples of heat-inactivated bacteria and sterile medium (Figure S5).

### Antimicrobial susceptibility assessment using dextran-coated magnetic nanosensors

**Figure 3:**
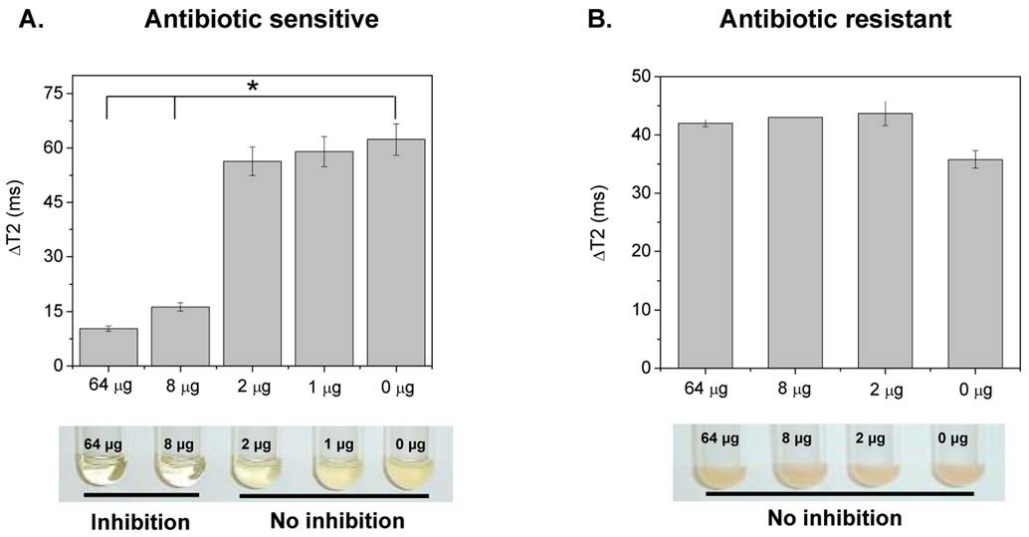
Antimicrobial susceptibility screening in MH broth with dextran-coated polysaccharide nanosensors. (A) Determination of *E. coli*'s minimum inhibitory concentration in MH broth, using the changes in spin-spin relaxation times (ΔT2, upper panel) (Means±SE; p<0.05) and the turbidity method (lower panel). (B) Identification of *S. marcescens*' resistance to ampicillin via ΔΤ2 (upper panel, Means±SE) and the turbidity assay (lower panel). The corresponding amount of ampicillin is indicated in the graphs and pictures of the bacterial cultures.

Based on our dextran-coated magnetic nanosensors' ability to sense carbohydrate utilization due to microbial metabolism, we examined if these nanosensors can be used for the identification of antimicrobial susceptibility and determination of an antibiotic's minimum inhibitory concentration (MIC). As MIC predicts the success of a particular antibiotic and is an important clinical parameter that dictates treatment in order to minimize adverse side effects, such as renal failure, quick determination of MIC is of paramount importance [5]. Thus, we first examined if the presence of antibiotics in the media might induce non-specific nanoparticle clustering, potentially interfering with the assay's carbohydrate specificity. In these control studies, a bacterial population (10^6^ CFU) which is typically used in MIC experiments was incubated in the presence of different antibiotic concentrations. Results showed that these culture conditions did not affect the spin-spin relaxation times of the nanoparticle solution, indicating absence of non-specific antibiotic-mediated nanoparticle assembly (Figure S6). Subsequently, we examined if bacterial growth and the corresponding carbohydrate uptake are affected by the presence of a particular antibiotic. After incubating *E. coli* for 2 hours in the presence of ampicillin, we took 10-µL bacterial culture aliquots in order to examine them using the dextran-coated polysaccharide nanosensors. Thirty minutes after addition of Con A distinct changes in the T2 were observed, indicating the presence of two cohorts (Figure 3A upper panel). Specifically, the starch utilization and growth of *E. coli* were suppressed at ampicillin concentrations above 8 µg, as demonstrated by the low changes in the ΔΤ2 compared to the sterile medium. Furthermore, the nanoparticle-derived MIC of 8 µg was confirmed through the turbidity method, which is the current gold standard for antimicrobial susceptibility determination (Figure 3A lower panel). Although both assays provided identical results, the dextran-coated polysaccharide nanosensor assay yielded faster results with an overall time of 2.5 hours (2 hours for bacterial-antibiotic incubation and 30 minutes for nanoparticle readout), as opposed to 24 hours. In addition, the nanosensor-based assay requires smaller bacterial culture volumes (10 µL) as opposed to the turbidity MIC method (2 mL). The latter is of particular logistics importance during epidemics and drug discovery efforts, as it facilitates the simultaneous and cost-effective screening of multiple samples, eliminating the need of tedious visual examination of numerous cultures.

Subsequently, we further validated the antimicrobial susceptibility potential of dextran-coated polysaccharide nanosensors using other bacteria. *Shigella sonnie*, a close relative of the highly pathogenic and Shiga-toxin producer *Shigella dysenteriae,* had an ampicillin MIC of 8 µg (Figure S7). Similar to *E. coli*, these results were also obtained within 2.5 hours. Then, we investigated if our assay can determine bacterial drug resistance via the changes in spin-spin relaxation time. As a model system, we used the hospital-acquired pathogen *Serratia marcescens* [25]. This pathogen is resistant to many antibiotics, including ampicillin, due to the presence of resistance plasmids, and is used as a model organism for bacterial drug resistance [26], [27]. Thirty minutes after addition of Con A into the nanoparticle solution, all samples exhibited similar ΔΤ2 values with no statistically significant differences, indicating that this pathogen is not susceptible to ampicillin (Figure 3B upper panel). Confirmation of our results was achieved via the turbidity method, where after 24 hours all cultures had a reddish turbid appearance, due to the characteristic production of the pigment prodigiosin by *S. marcescens* [28] (Figure 3B lower panel).

**Figure 4:**
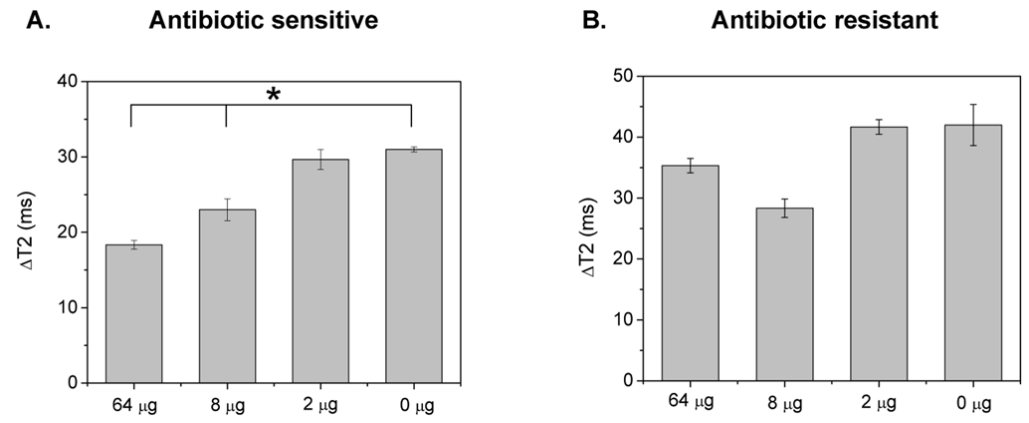
Dextran-coated polysaccharide nanosensor-mediated determination antimicrobial susceptibility in blood. (A) Assessment of *E. coli*'s ampicillin MIC, and (B) identification of *S. marcescens'* ampicillin resistance in blood (Means±SE; p<0.05).

Due to the fact that many bacteria can either cause septicemia or require growth in optically turbid media, it is important to assess bacterial susceptibility in these complex matrices. However, most current methods cannot be utilized for the detection of molecular targets and assessment of antimicrobial susceptibility in blood, due to the strong absorbance and scattering from the matrix's constituents, including platelets and red blood cells (Figures S8 and S9). Therefore, considering these drawbacks and the facts that bacterial isolation is a major limitation step in diagnosis and that certain pathogenic microorganisms require growth in specialized media, we investigated if the dextran-coated polysaccharide nanosensors can assess antimicrobial susceptibility in blood. Recently, we reported the high-throughput bacterial susceptibility determination, using the surface plasmon band shifts of gold nanoparticles [12]. However, this method cannot be used in opaque media, such as blood, due to the matrix's intrinsic optical properties, masking the nanoparticles' plasmonic band (Figure S8). To investigate this, we used *E. coli* and *S. marcescens* cultures in blood-supplemented MH broth, grown in the presence of ampicillin for 2 hours at 37°C. Aliquots of these cultures (10 µL) were obtained and added into the dextran-coated polysaccharide nanosensors working solution, followed by 10-µL Con A treatment (1 µg/µL). After 45 minutes post-Con A addition at room temperature, we determined that *E. coli*'s ampicillin MIC was 8 µg (Figure 4A), without observing any nanoparticle precipitation (Figure S10). Additionally, the *S. marcescens*' drug resistance was identified after an hour-long incubation at 25°C (Figure 4B).

### Antimicrobial susceptibility assessment using Concanavalin A-conjugated polysaccharide nanosensors

**Figure 5:**
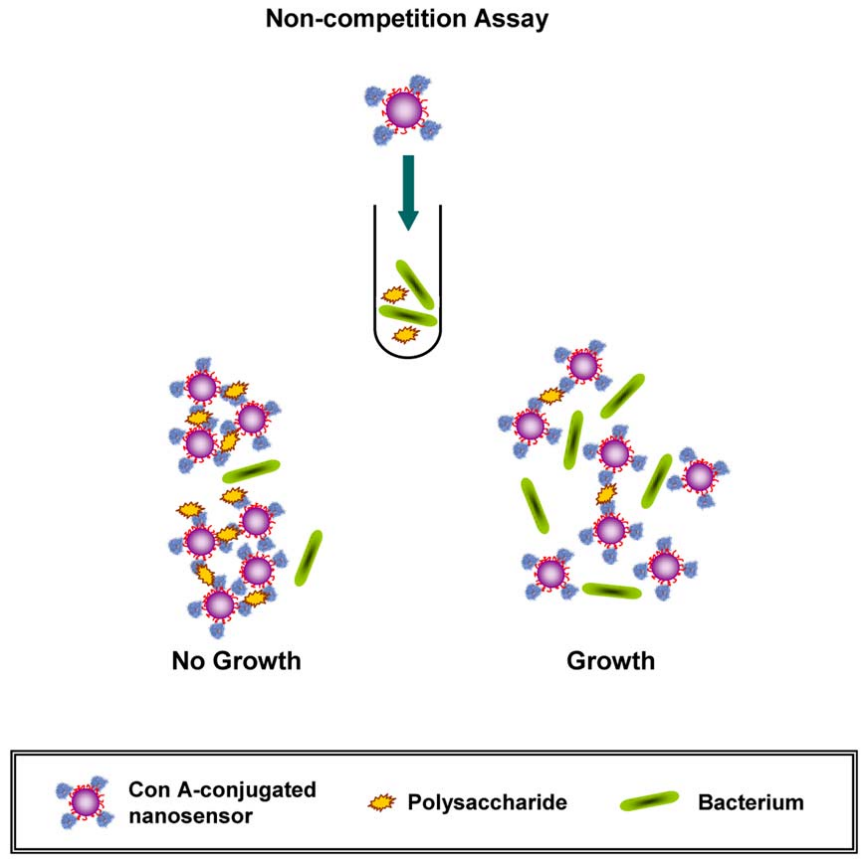
Schematic representation of the assessment of antimicrobial susceptibility using Concanavalin A-conjugated polysaccharide nanosensors. The use of silica-coated nanoparticles and the direct conjugation of Con A to the capping matrix result in a non-competition-based assay format, which may potentially provide faster readout times.

Often times a slight modification in the nanosensors' design and/or the protocol followed can result in significant improvements in either the sensitivity or speed of the assay [29], [30]. Therefore, we hypothesized whether conjugating Con A to the surface of the magnetic nanoparticles would allow for faster kinetics and shorter the detection time. For these experiments, we conjugated Con A directly to aminated silica-coated iron oxide nanoparticles. We chose silica-coated instead of dextran-coated iron oxide nanoparticles to avoid possible cross reaction with the dextran on the nanoparticle's surface. In this non-competition assay (Figure 5), the Con A-conjugated silica coated nanosensors would facilitate the direct sensing of the levels of carbohydrates in solution, as opposed to the competition assay that requires two reagents; the dextran-coated nanoparticles and the Con A for successful sensing. The aminated silica-coated nanoparticles were synthesized using a modified water-based synthetic protocol [31]. The resulting nanoparticles were monodispersed, having a diameter of 145 nm (Figure S11A) and an R2 relaxivity of 225 mM^−1^s^−1^.

**Figure 6:**
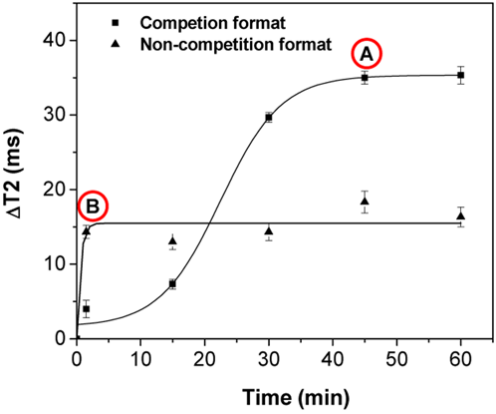
Kinetic profiles of the competition and non-competition assay formats. Point A represents the end-point of the competition assay utilizing the dextran-coated polysaccharide nanosensors, whereas Point B corresponds to the end-point of the non-competition assay based on the Con A-conjugated polysaccharide nanosensors.

In our first set of experiments with the aminated silica-coated nanoparticles, we determined whether these nanoparticles clustered non-specifically in the presence of Con A in solution. As expected, we observed that Con A did not induce any changes in the relaxation times of the nanoparticles (Figure S12). This demonstrates that the silica coating on these nanoparticles lacks any carbohydrate epitopes, rendering them suitable for the non-competition-based sensing of carbohydrates. Therefore, we conjugated Con A to the aminated silica-coated nanoparticles, via carbodiimide chemistry, resulting in Con A-carrying nanoparticles with a hydrodynamic diameter of ∼160 nm (Figure S11B) (R2 = 225 mM^−1^s^−1^, [Con A] = 0.03 µg/µL). First, we compared the kinetic profiles of the dextran-coated nanosensors and Con A-conjugated nanosensors using bacterial *E. coli* blood cultures (10^6^ CFU grown in the presence of 2 µg ampicillin). Interestingly, we found that the non-competition assay with the Con A-conjugated nanosensors (Figure 6, curve B) provided faster results than the competition assay that utilizes the dextran-coated nanosensors (Figure 6, curve A). Specifically, the competition assay format reached its end-point after a 45-minute incubation, whereas the non-competition assay reaches its end-point within 5 minutes upon addition of the bacterial sample. These findings support our hypothesis of achieving faster kinetics due to the direct conjugation of Con A to the nanoparticles.

**Figure 7:**
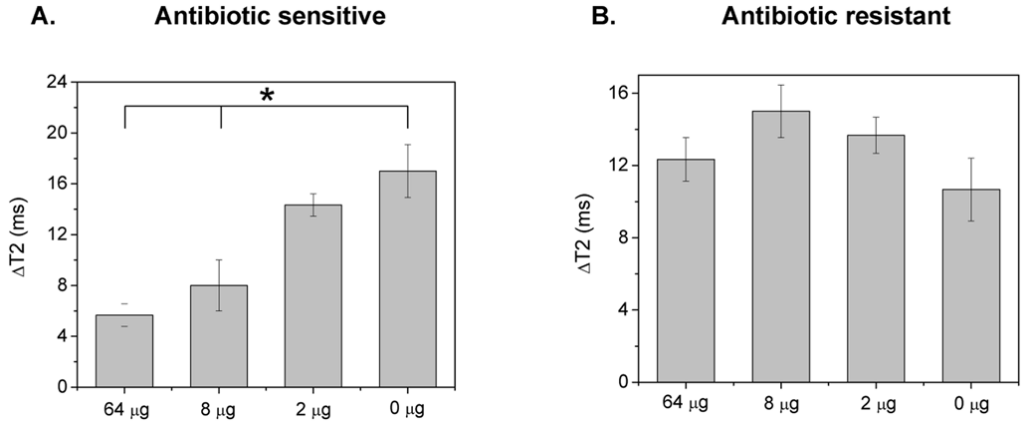
Antimicrobial susceptibility in blood using Con A-conjugated polysaccharide nanosensors. (A) Determination of *E. coli*'s ampicillin MIC in blood, and (B) determination of *Serratia marcescens*' drug resistance in blood with the Con A-conjugated polysaccharide nanosensors, five minutes after addition of the bacterial aliquot into the nanoparticle solution (Means±SE; p<0.05).

Then, we examined if MIC determination can be achieved using these Con A-conjugated nanosensors, in blood cultures of *E. coli* and *S. marcescens*. Immediately upon addition of the bacterial sample into the nanoparticle solution, distinct changes in the T2 were observed. Specifically, within 5 minutes the Con A-nanosensors were able to determine that *E. coli* had an ampicillin MIC of 8 µg (Figure 7A), in line with the data from the dextran-coated nanosensors in the competition assay or the turbidity test. Likewise, within 5 minutes the Con A-conjugated nanosensors assessed that *S. marcescens* was resistant to ampicillin, further corroborating the findings that the non-competition assay format provides faster results than the competition-based assay of the dextran-coated nanosensors (Figure 7B).

## Discussion

Concluding, we have shown that superparamagnetic iron oxide nanoparticles, either dextran-coated supplemented with Con A or Con A-conjugated nanosensors, can be used for the quantification of polysaccharides, assessment of metabolic activity and determination of antimicrobial susceptibility in complex matrices, such as blood, through magnetic relaxation. This approach outperforms optical-based assays, which cannot be utilized in opaque media. Additionally, both iron oxide nanoparticle assay formats require very small sample volumes, are equally reliable to gold standard methods, and do not require any sample preparation, in contrast to other techniques [32]. Notably though, the Con A-conjugated polysaccharide nanosensor assay yields faster results, without compromising sensitivity and reliability, due to faster binding kinetics. Also, as there is no need for the addition of a second reagent (Con A), this format might be particularly useful for point-of-care diagnostics and applications in the field. Furthermore, the iron oxide nanoprobes can be easily adapted for the high-throughput screening of multiple clinical and environmental samples, preventing epidemics and promoting drug discovery. In view of the recent advancements in NMR technology [33], [34], [35], the high-throughput and/or field analysis of multiple samples in complex media should be feasible, even outside of the typical laboratory setting. Overall, we believe that these assays can expedite clinical decision-making and may facilitate the faster discovery of potential antimicrobial agents. Further studies are in progress to determine microbial susceptibility to antifungal and antibacterial agents in clinical samples and other matrices.

## Materials and Methods

### Synthesis of iron oxide nanoparticles

#### Reagents

All the reagents used were of AR (Analytical Reagent) grade. Nitrogen-purged double-distilled water was used throughout the reaction. Iron salts, FeCl_2_.4H_2_O and FeCl_3_.6H_2_O, were obtained from Fluka. Dextran (MW 10kDa) was received from Amersham. TEOS: tetraethylorthosilicate (Fluka), APTS: 3-(amino-propyl)triethoxysilane (Aldrich) and THPMP: 3-(trihydroxysilyl)propylmethyl-phosphonate (Gelest Inc) were used as received from the suppliers.

#### Procedure

The dextran-coated iron oxide nanoparticles were prepared as previously reported in the literature [16]. The aminated silica-coated iron oxide nanoparticles were prepared using a previously published protocol [31], with modifications in order to yield stable nanoparticles via a water-based synthesis. Specifically, iron oxide nanocrystals were formed via the alkaline precipitation method, by mixing a solution of iron salts (0.202 g FeCl_2_.4H_2_O, 0.488 g FeCl_3_.6H_2_O, 88.7 µL HCl in 2 mL distilled water) with an ammonium hydroxide solution (830 µl NH_4_OH in 15 mL distilled water). Then, 20 seconds after the initiation of the iron oxide nanocrystal formation, a TEOS-THPMP-APTS solution was added (6180 µL THPMP, 2680 µL TEOS, 670 µL APTS) under continuous vortexing. The as-synthesized nanoparticle suspensions were centrifuged to remove large particles. Both the amino-silica- and dextran-coated nanoparticles were washed several times with distilled water and concentrated through an Amicon 8200 cell (Millipore Ultrafiltration membrane YM–30 k). Finally, the nanoparticle suspensions were stored at 4°C until further use.

#### Conjugation of Concanavalin A to aminated silica-coated iron oxide nanoparticles

Two milliliters of aminated silica-coated iron oxide nanoparticles (R2 = 225 mM^−1^s^−1^, [Fe] = 0.47 mg/ml) were used for the conjugation of Con A to the nanoparticles' surface. Initially, in 1 mL of cold MES buffer (0.1 M, pH 6.0) 4.8 mg EDC (Pierce) and 3 mg NHS (Pierce) were dissolved. Then, 2 mg of lyophilized Con A (Type V, Sigma) were dissolved in 2 mL cold MES buffer (0.1 M, pH 6.0). Subsequently, the Con A solution was mixed with the EDC/NHS solution, followed by a 3-minute low-speed rotary mixing at room temperature. Finally, the aminated silica-coated iron oxide nanoparticles were added to the Con A (amine-reactive NHS-ester form) solution, followed by periodical rotary mixing at low speed and storage at 4°C. The resulting Con A-conjugated silica-coated iron oxide nanoparticles were purified from any unbound protein via magnetic separation using an MES buffer-equilibrated (0.1 M) LS25 MACS® column (Miltenyi Biotec).

### Characterization

Dynamic light scattering (DLS) studies were done using a PDDLS CoolBatch 40T instrument using Precision Deconvolve 32 software. Iron concentration was determined spectrophotometrically after acid digestion of the nanoparticles' suspension, whereas R2 relaxivity measurements were obtained using a 0.47T mq20 NMR analyzer (Minispec, Bruker, Germany). Starch sensing with the magnetic nanoparticles was performed using serial dilutions of starch (∼75% amylopectin, ∼25% amylose, S-516, Fisher) in non-starch containing MH broth (DIFCO™, BD). The concentration of Con A conjugated to the silica-coated iron oxide nanoparticles was determined through the BCA assay, in accordance to the manufacturer's protocol (Pierce), after magnetic separation of the nanoparticle suspension using an MES buffer-equilibrated (0.1 M) LS25 MACS® column (Miltenyi Biotec).

### Bacterial cultures

In order to investigate if the dextran-coated iron oxide nanoparticles can monitor the starch utilization due to bacterial metabolic activity, different populations of *Escherichia coli* (strain 8739 from ATCC) were grown in starch-containing MH broth (DIFCO™, BD) for 2 hours at 37°C. For determination of the minimum inhibitory concentration, *Escherichia coli* (10^6^ CFU), *Serratia marcescens* (ATCC, 10^6^ CFU) and *Shigella sonnie* (strain 9290 from ATCC, 10^6^ CFU) were grown in a starch-containing MH broth (DIFCO™, BD), for 2 hours at 37°C in the presence or absence of ampicillin. For determination of MIC in blood, bacterial stocks (10^6^ CFU) were grown in the presence or absence of ampicillin in a 5%-blood-supplemented starch-containing MH broth, for 2 hours at 37°C. Defibrinated sheep blood was obtained from the Colorado Serum Company, simulating bacterial isolation and growth in typical blood agar plates. For studies requiring heat inactivation, *E. coli* bacteria were autoclaved in the culture tubes for 10 minutes. Upon incubation or inactivation, all bacterial stocks were placed in a Fisher Isotemp freezer (Fisher Scientific, Hampton, NH), until further use.

### Sample preparation for relaxation measurements

Aliquots of 10 µl (either of the aforementioned bacteria or media) were added into 190 µl nanoparticle working solution (0.02 µg/µL in Ca^2+^/Mg^2+^-free 1× PBS for both the dextran-coated and Con A-conjugated polysaccharide nanosensors). For the dextran-coated polysaccharide nanosensors, 10 µL Con A (Sigma, 1 µg/µl) were added in order to induce nanoparticle clustering.

### Measurement of proton relaxation times

Spin-spin relaxation times (T2) were measured using a 0.47 T mq20 NMR analyzer (Minispec, Bruker, Germany). T2 values were obtained before and after addition of the aliquot, and through the time course of the study. For the quantification of starch, we prepared serial dilutions of sterile starch-supplemented MH broth (DIFCO™, BD) in sterile non-starch-supplemented MH broth (DIFCO™, BD). Subsequently, the dextran-coated polysaccharide nanosensors' sensitivity and dose response to the analyte (starch) was evaluated, by monitoring the changes in the spin-spin relaxation time. In this case, ΔΤ2 is denoted as the difference between the relaxation time of the solution's initial dispersed state before addition of the MH broth aliquot (T2_in_) and the solution's T2 after addition of the MH broth aliquot (T2′). Thus, ΔT2 = |T2′−T2_initial_|. For all other measurements, including *E.coli*'s metabolic activity monitoring via carbohydrate quantification and magnetic relaxation-mediated antimicrobial susceptibility assessment, ΔΤ2 is denoted as the difference between the T2 of a sample and the corresponding sterile control sample (ΔΤ2 = |Τ2_sample_−T2_control_|), where the sterile control was starch-containing MH which is used for the growth of these microorganisms. The sterile control used for the nanoparticle-mediated antimicrobial susceptibility assessment in blood was 5%-blood-supplememted starch-containing MH broth. All experiments and measurements were carried out in triplicate and data were expressed as mean±standard error, unless otherwise denoted. Response curves were obtained by fitting the data on a sigmoidal curve, using Origin 7.5 (OriginLab, Northampton, MA). Statistical analyses were performed on SPSS 11.5 (SPSS Inc., Chicago, IL).

### Broth dilution assay for determination of Minimum Inhibitory Concentration (MIC)

Broth dilution assay (standard method for measuring MIC) was achieved by inoculating serial dilutions of ampicillin in sterile starch-containing MH broth, with either *E. coli*, *S. marcescens*, or *S. sonnei* (10^6^ CFU), followed by a 24-hour long incubation at 37°C. Bacterial growth was assessed based on the broth's turbidity, where absence of turbidity was an indicator of successful antimicrobial susceptibility. The lowest ampicillin concentration where the MH broth is clear indicates the minimum concentration of ampicillin that can successfully inhibit bacterial growth [4], [5].

## Supporting Information

Figure S1 Size distribution of dextran-coated polysaccharide nanosensors **(A)** before addition of Con A, and **(B)** one hour after Con A addition (1 µg/µl), indicating the formation of nanoparticle clusters.
(0.30 MB TIF)

Figure S2 Treatment with 10 µl of Con A (1 µg/µl) facilitates the clustering of dextran-coated iron oxide nanoparticles, resulting in prominent changes in the solution's spin-spin relaxation time (T2).
(0.24 MB TIF)

Figure S3 Standard curve for determination of Con A's optimum concentration using a nanoparticle solution with a concentration of 0.02 µg Fe/µl.
(0.24 MB TIF)

Figure S4 In the absence of ConA, the IO NPs were in a non-assembled state, exhibiting the same T2 regardless of the presence of bacteria, after a 30-minute incubation. **1**. Water, **2**. Sterile medium (with starch), **3**. Sterile medium (no starch), **4**. 10^2^ CFU *E. coli*, **5**. 10^3^ CFU *E. coli*, **6**. 10^4^ CFU *E. coli*, **7**. 10^5^ CFU *E. coli*, **8**. 10^6^ CFU *E. coli*, **9**. 10^8^ CFU *E. coli*, **10**. 10^9^ CFU *E. coli.*
(0.29 MB TIF)

Figure S5 The behavior of IO NPs is independent of the heat-inactivated bacterial population, but it is dependent of active bacterial metabolism. The graph depicts data obtained after a 30-minute incubation at room temperature, in the presence of Con A. Linear fit was applied with an R^2^ = 0.14 (OriginPro 7.5). Similar results were obtained after 0-, 90- and 150-minute incubations.
(0.25 MB TIF)

Figure S6 In the absence of ConA, the IO NPs exhibited the same T2, regardless of the presence of bacteria and antibiotic. **1**. Water, **2**. Sterile medium (with starch), **3**. Sterile medium (no starch), **4**. 64 µg ampicillin, **5**. 8 µg ampicillin, **6**. 2 µg ampicillin, **7**. 1 µg ampicillin, **8**. 0 µg ampicillin.
(0.28 MB TIF)

Figure S7 Determination of the minimum inhibitory concentration of *Shigella sonnie* using the changes in spin-spin relaxation times (ΔT2) after a 30-min incubation at 25° (Means ± SE; p <0.05). The dotted line indicates the threshold of the drug's successful inhibition.
(0.41 MB TIF)

Figure S8 UV-vis profile of a bacterial culture growing in 5%-blood-supplemented MH broth. *E. coli* (10^6^ CFU) were incubated for 2 hours at 37° in the presence of 4 µg ampicillin, in 5%-blood-supplemented starch-containing MH broth. Then, a 50-µl bacterial culture aliquot was diluted in 950 µl 1× PBS (Ca^2+^/Mg^2+^-free), resulting in similar to the relaxation setup bacterial aliquot dilution. The strong absorbance of blood in the visible spectrum suggests that optical-based methods spanning from 400 to 800 nm may not be effective for MIC determination in this matrix.
(0.25 MB TIF)

Figure S9 Size distribution of **(A)** dextran-coated gold nanoparticles and **(B)** 5%-blood-supplemented MH broth, indicating that the clustering of dextran-coated gold nanoparticles cannot be used for antimicrobial susceptibility assessment in blood.
(0.30 MB TIF)

Figure S10 Blood cultures in nanoparticle solution demonstrating the absence of nanoparticle precipitation and the optical nature of the solution (numbers indicate the corresponding concentration of ampicillin in µg).
(0.36 MB TIF)

Figure S11 Size distribution of silica-IO nanoparticles **(A)** prior and **(B)** after Con A conjugation.
(0.29 MB TIF)

Figure S12 Absence of Con A-induced clustering in silica-coated IO nanoparticles, indicating the lack of carbohydrate-containing moieties on the nanoparticles' surface.
(0.29 MB TIF)
