# Supplementary figures and images for: Rapid Nanoparticle-Mediated Monitoring of Bacterial Metabolic Activity and Assessment of Antimicrobial Susceptibility in Blood with Magnetic Relaxation
Source: PLoS One. 2008 Sep 23;3(9):e3253. doi: 10.1371/journal.pone.0003253 (PMC2533125; doi:10.1371/journal.pone.0003253)

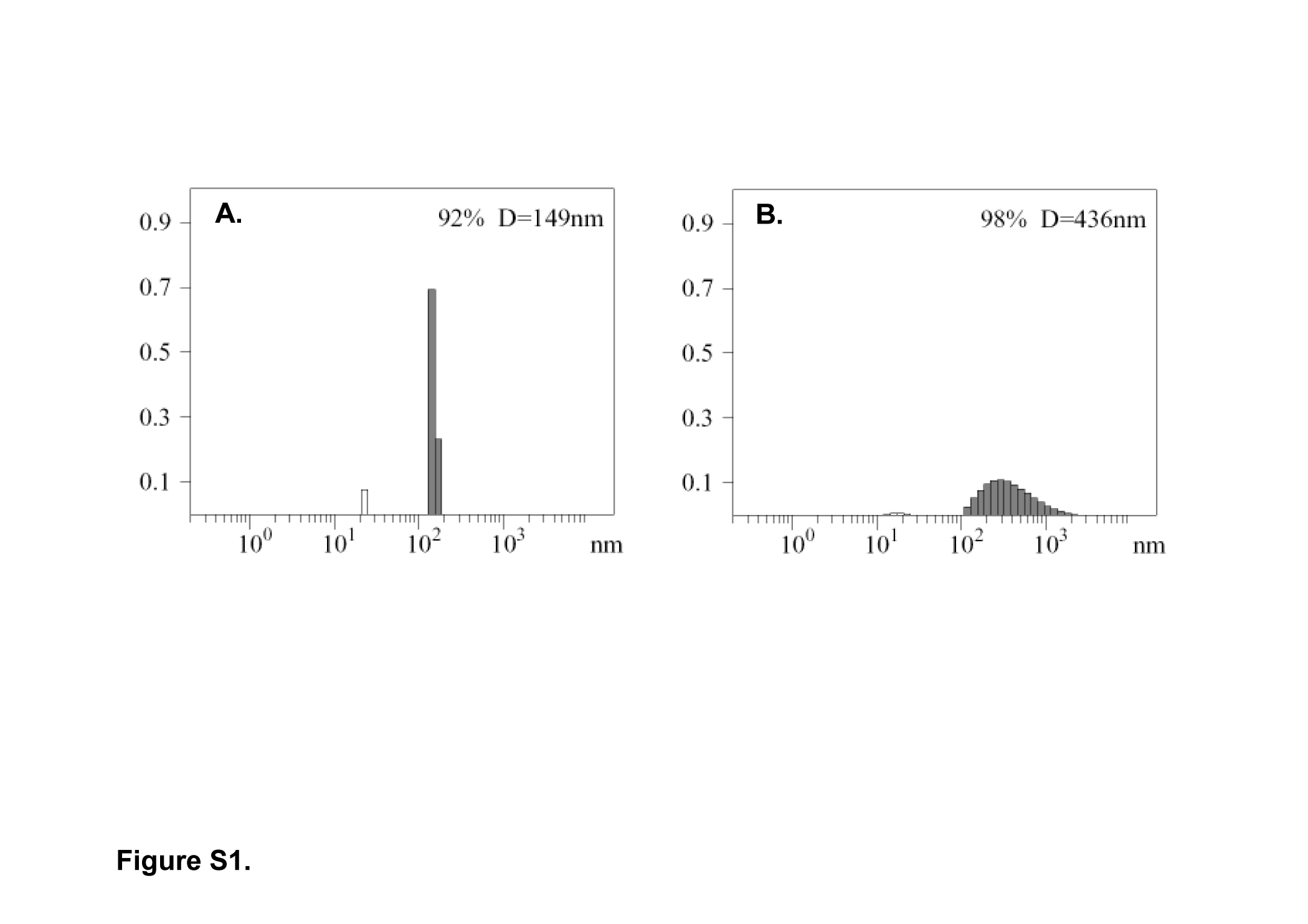

Supplement: Figure S1 — Size distribution of dextran-coated polysaccharide nanosensors (A) before addition of Con A, and (B) one hour after Con A addition (1 µg/µl), indicating the formation of nanoparticle clusters. (0.30 MB TIF) [file pone.0003253.s001.tif]

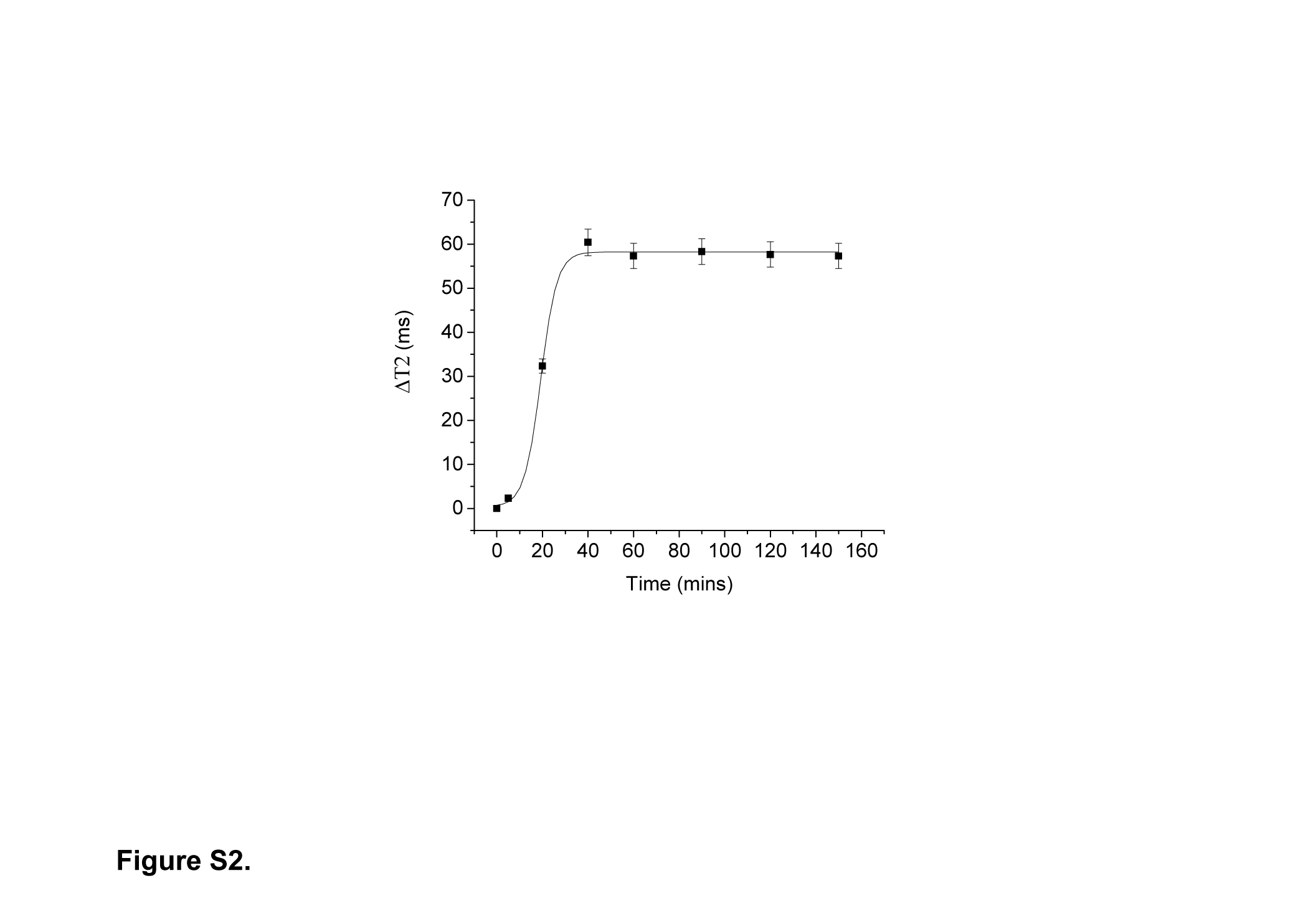

Supplement: Figure S2 — Treatment with 10 µl of Con A (1 µg/µl) facilitates the clustering of dextran-coated iron oxide nanoparticles, resulting in prominent changes in the solution's spin-spin relaxation time (T2). (0.24 MB TIF) [file pone.0003253.s002.tif]

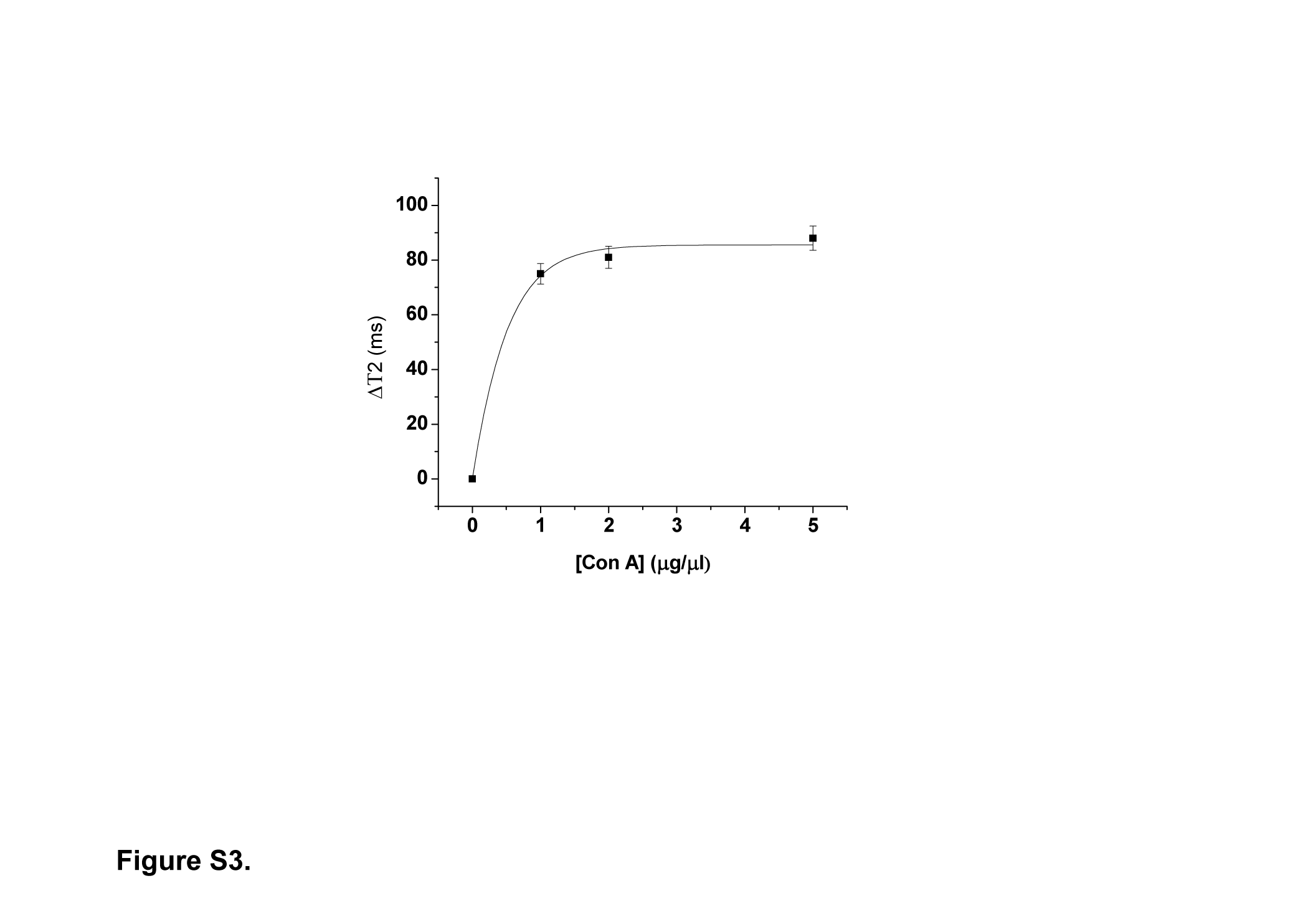

Supplement: Figure S3 — Standard curve for determination of Con A's optimum concentration using a nanoparticle solution with a concentration of 0.02 µg Fe/µl. (0.24 MB TIF) [file pone.0003253.s003.tif]

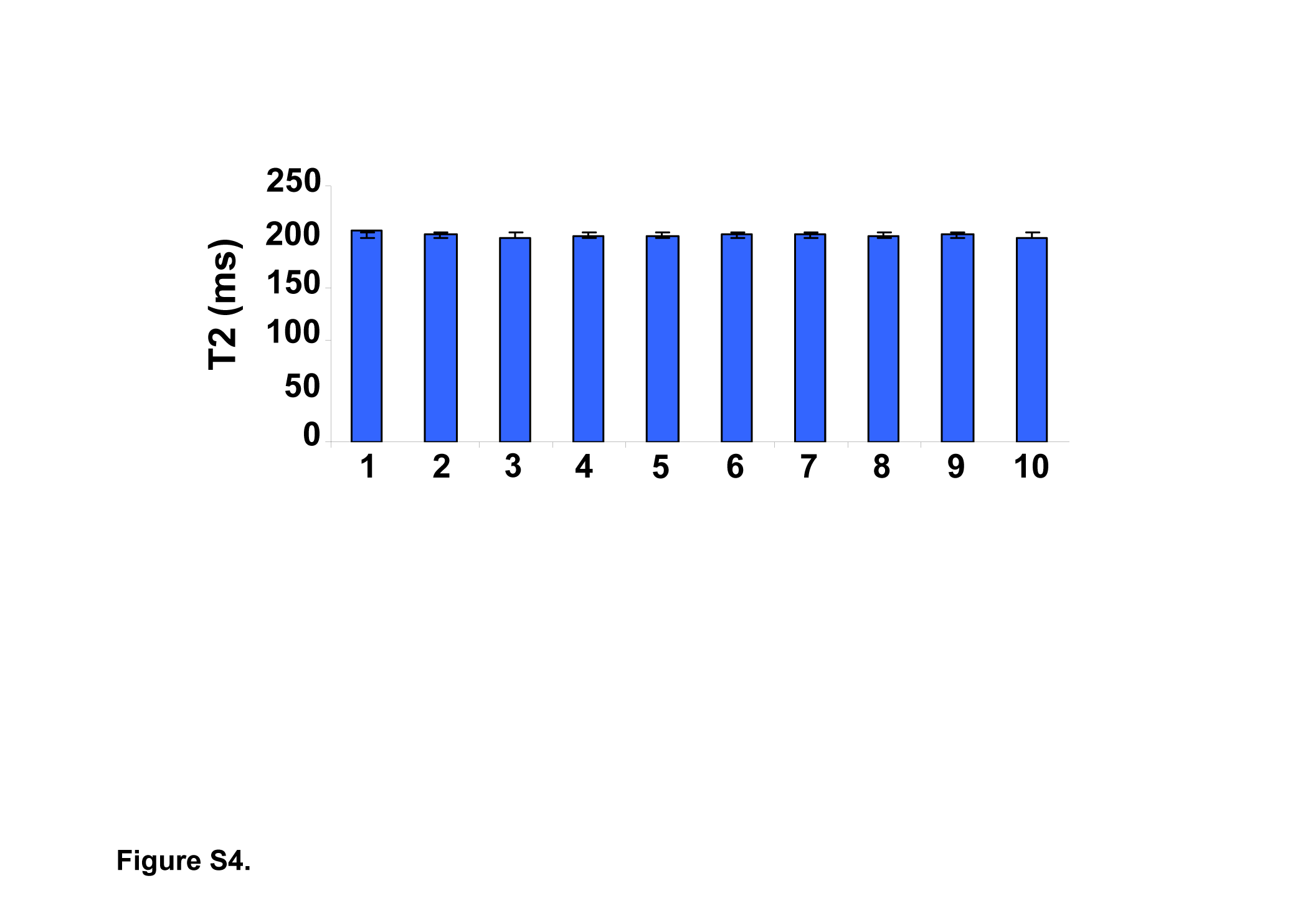

Supplement: Figure S4 — In the absence of ConA, the IO NPs were in a non-assembled state, exhibiting the same T2 regardless of the presence of bacteria, after a 30-minute incubation. 1. Water, 2. Sterile medium (with starch), 3. Sterile medium (no starch), 4. 102 CFU E. coli, 5. 103 CFU E. coli, 6. 104 CFU E. coli, 7. 105 CFU E. coli, 8. 106 CFU E. coli, 9. 108 CFU E. coli, 10. 109 CFU E. coli. (0.29 MB TIF) [file pone.0003253.s004.tif]

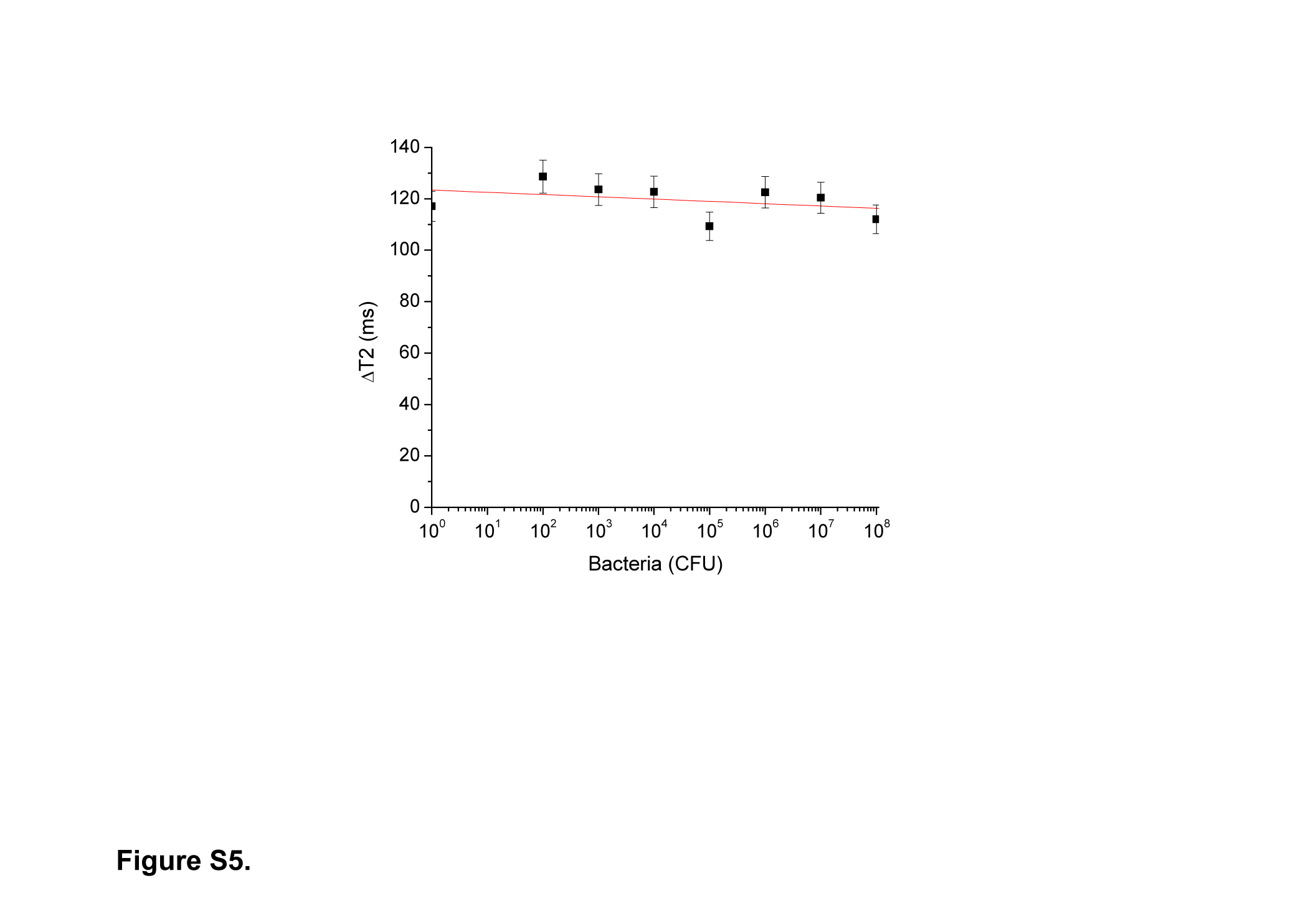

Supplement: Figure S5 — The behavior of IO NPs is independent of the heat-inactivated bacterial population, but it is dependent of active bacterial metabolism. The graph depicts data obtained after a 30-minute incubation at room temperature, in the presence of Con A. Linear fit was applied with an R2 = 0.14 (OriginPro 7.5). Similar results were obtained after 0-, 90- and 150-minute incubations. (0.25 MB TIF) [file pone.0003253.s005.tif]

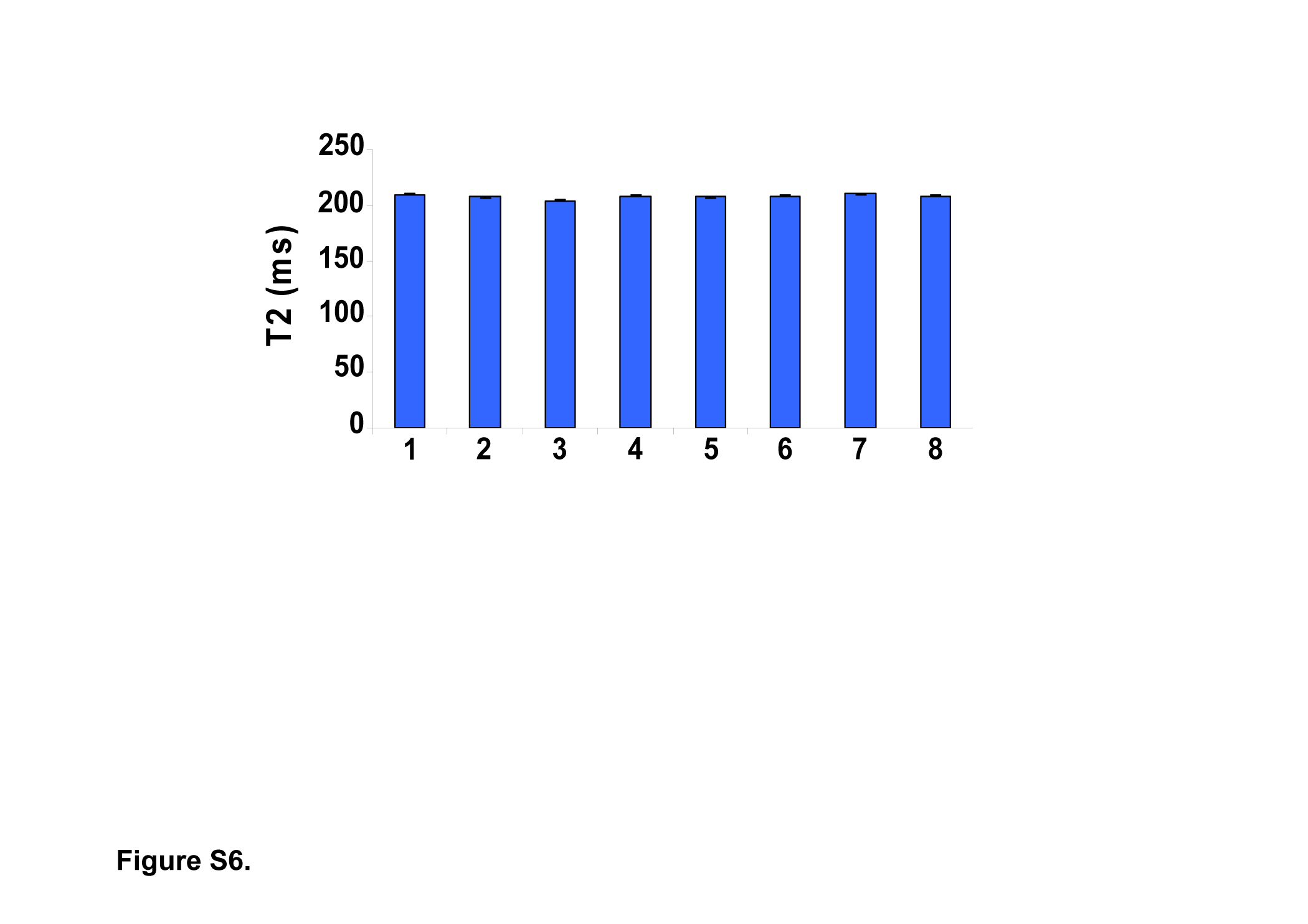

Supplement: Figure S6 — In the absence of ConA, the IO NPs exhibited the same T2, regardless of the presence of bacteria and antibiotic. 1. Water, 2. Sterile medium (with starch), 3. Sterile medium (no starch), 4. 64 µg ampicillin, 5. 8 µg ampicillin, 6. 2 µg ampicillin, 7. 1 µg ampicillin, 8. 0 µg ampicillin. (0.28 MB TIF) [file pone.0003253.s006.tif]

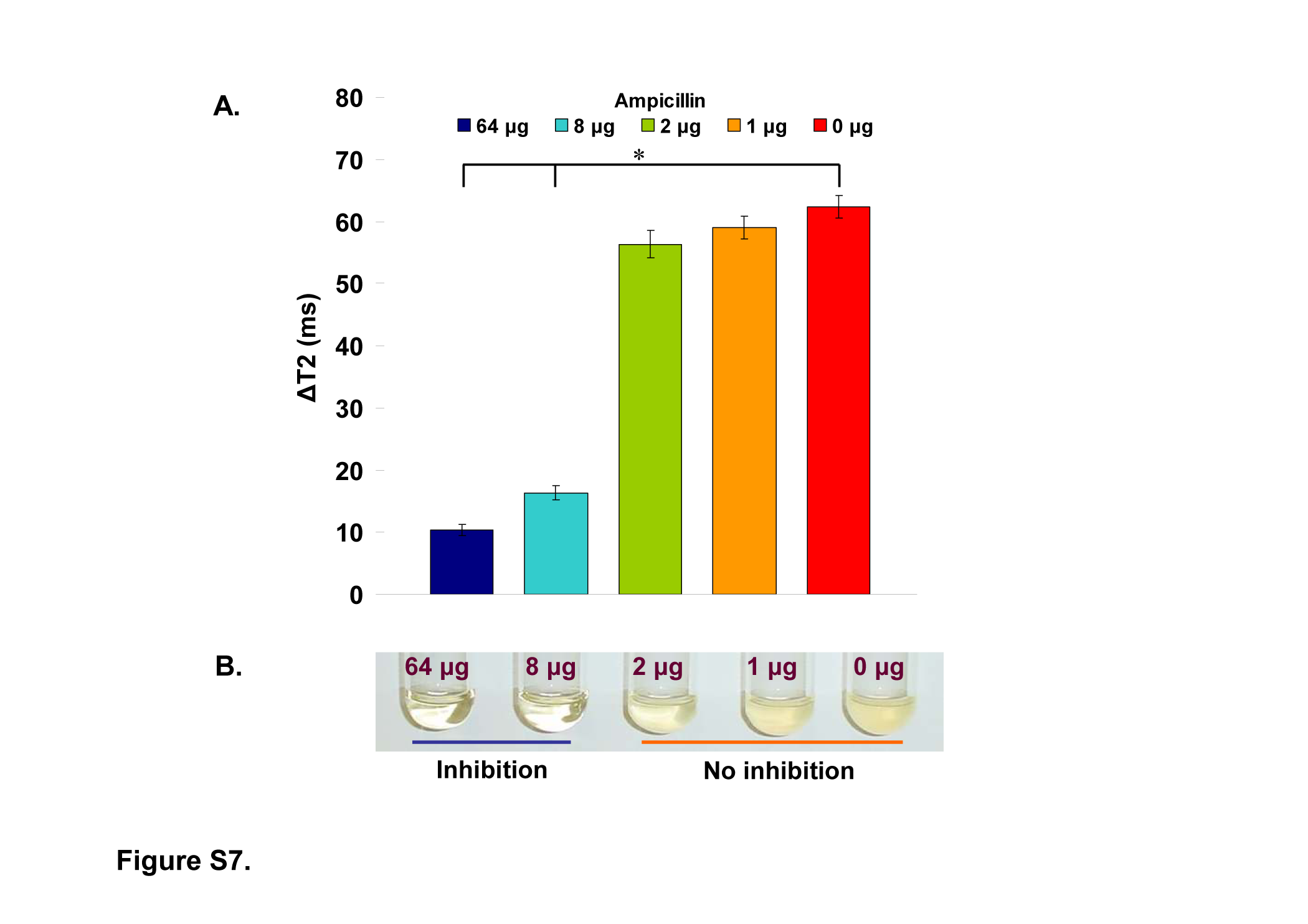

Supplement: Figure S7 — Determination of the minimum inhibitory concentration of Shigella sonnie using the changes in spin-spin relaxation times (ΔT2) after a 30-min incubation at 25° (Means ± SE; p <0.05). The dotted line indicates the threshold of the drug's successful inhibition. (0.41 MB TIF) [file pone.0003253.s007.tif]

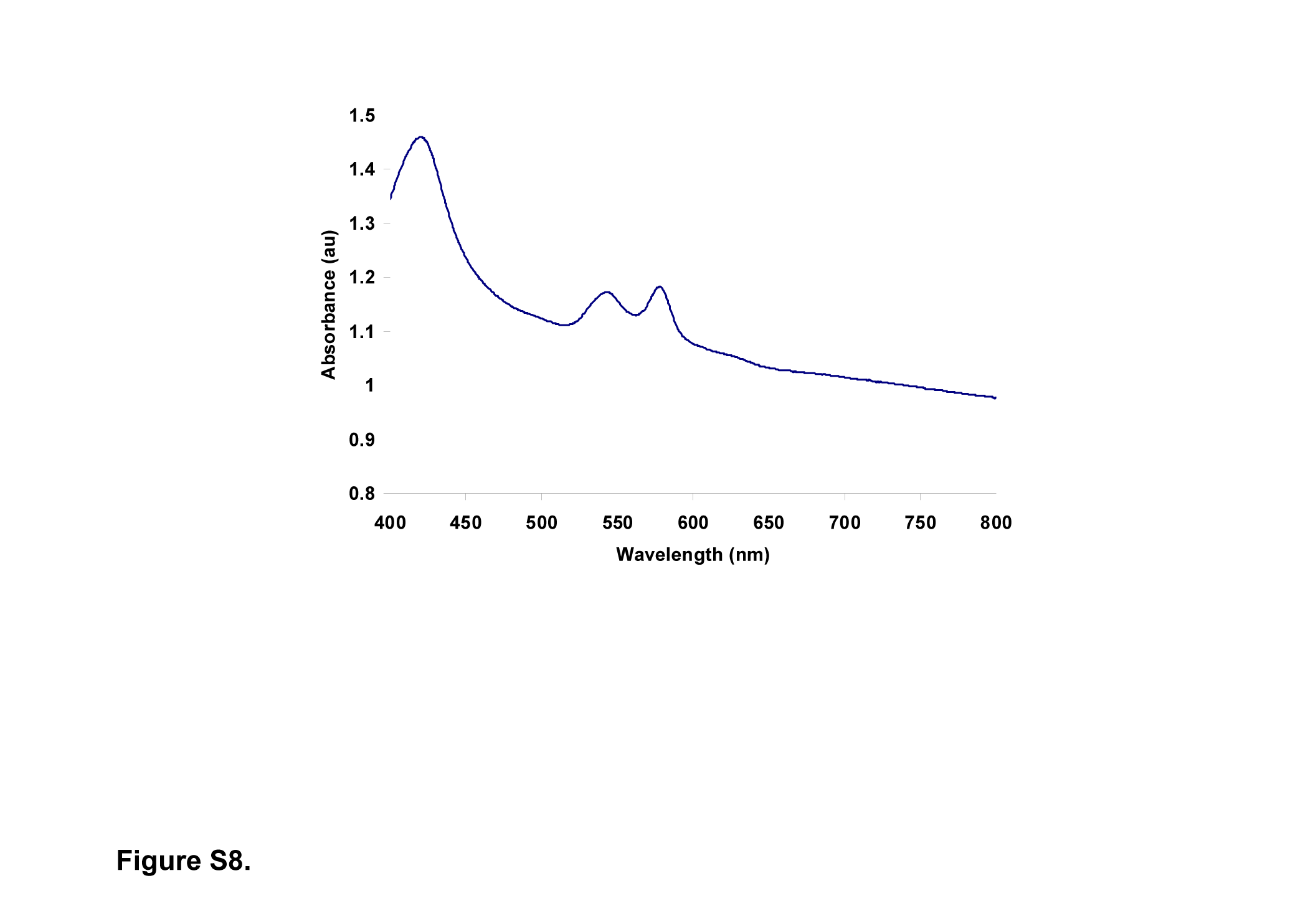

Supplement: Figure S8 — UV-vis profile of a bacterial culture growing in 5%-blood-supplemented MH broth. E. coli (106 CFU) were incubated for 2 hours at 37° in the presence of 4 µg ampicillin, in 5%-blood-supplemented starch-containing MH broth. Then, a 50-µl bacterial culture aliquot was diluted in 950 µl 1× PBS (Ca2+/Mg2+-free), resulting in similar to the relaxation setup bacterial aliquot dilution. The strong absorbance of blood in the visible spectrum suggests that optical-based methods spanning from 400 to 800 nm may not be effective for MIC determination in this matrix. (0.25 MB TIF) [file pone.0003253.s008.tif]

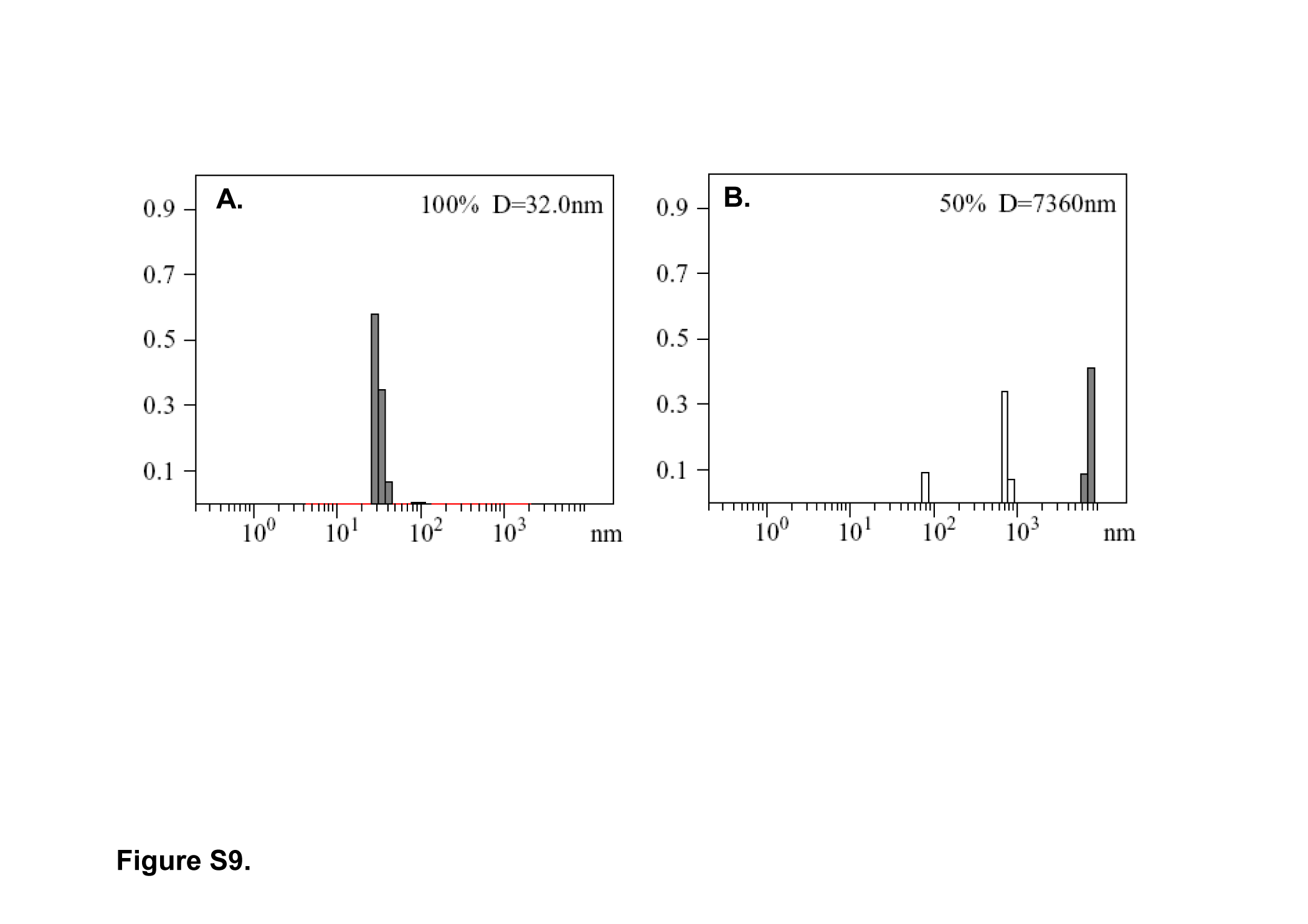

Supplement: Figure S9 — Size distribution of (A) dextran-coated gold nanoparticles and (B) 5%-blood-supplemented MH broth, indicating that the clustering of dextran-coated gold nanoparticles cannot be used for antimicrobial susceptibility assessment in blood. (0.30 MB TIF) [file pone.0003253.s009.tif]

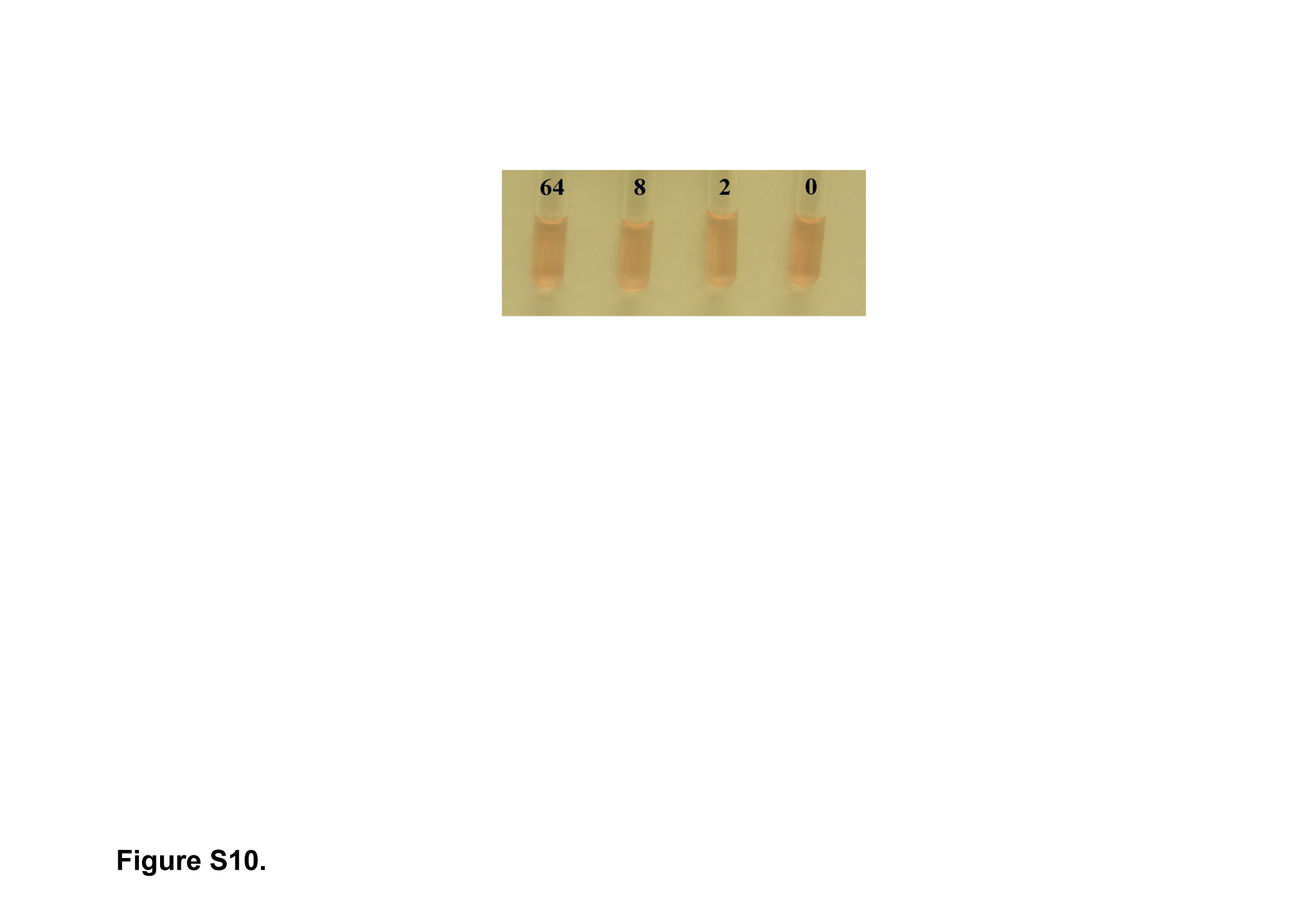

Supplement: Figure S10 — Blood cultures in nanoparticle solution demonstrating the absence of nanoparticle precipitation and the optical nature of the solution (numbers indicate the corresponding concentration of ampicillin in µg). (0.36 MB TIF) [file pone.0003253.s010.tif]

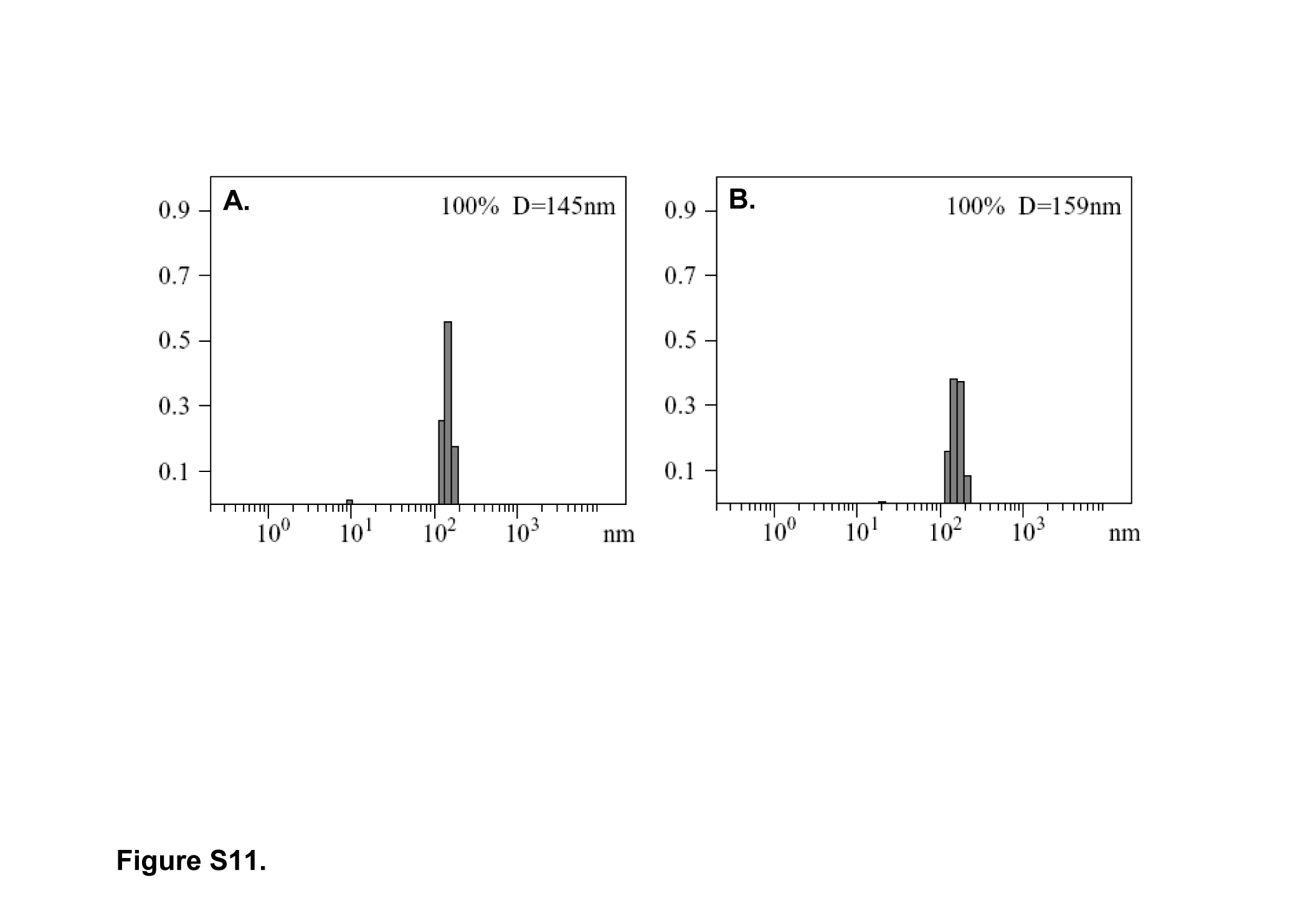

Supplement: Figure S11 — Size distribution of silica-IO nanoparticles (A) prior and (B) after Con A conjugation. (0.29 MB TIF) [file pone.0003253.s011.tif]

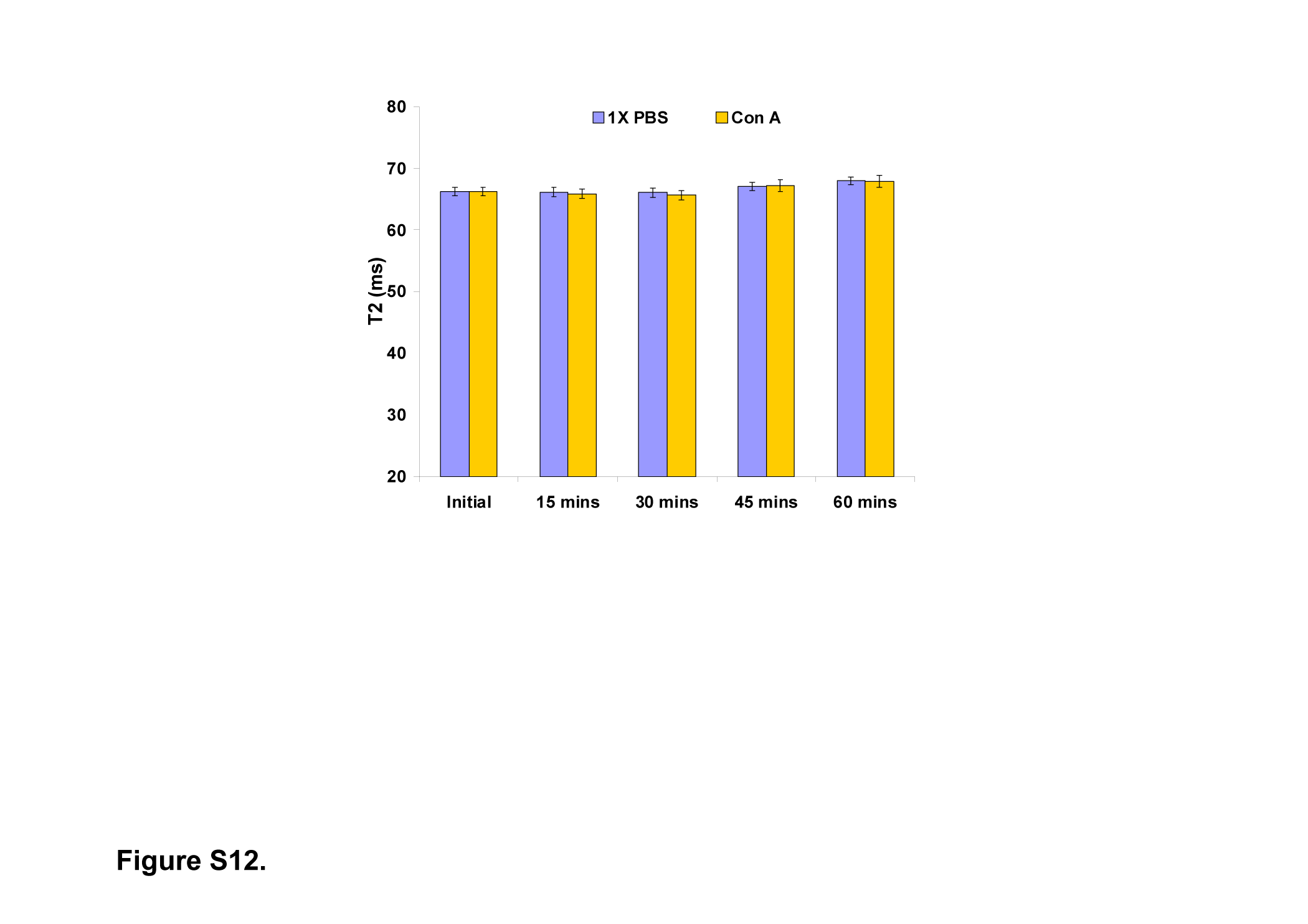

Supplement: Figure S12 — Absence of Con A-induced clustering in silica-coated IO nanoparticles, indicating the lack of carbohydrate-containing moieties on the nanoparticles' surface. (0.29 MB TIF) [file pone.0003253.s012.tif]
